# Development of a behaviour change intervention to increase upper limb exercise in stroke rehabilitation

**DOI:** 10.1186/s13012-015-0223-3

**Published:** 2015-03-12

**Authors:** Louise A Connell, Naoimh E McMahon, Judith Redfern, Caroline L Watkins, Janice J Eng

**Affiliations:** Clinical Practice Research Unit, School of Health, University of Central Lancashire, Preston, PR1 2HE England; Department of Physical Therapy, University of British Columbia, 212-2177 Wesbrook Mall, V6T 1Z3 Vancouver, British Columbia Canada

**Keywords:** Stroke, Upper limb, Behaviour change, Complex intervention, Development

## Abstract

**Background:**

Two thirds of survivors will achieve independent ambulation after a stroke, but less than half will recover upper limb function. There is strong evidence to support intensive repetitive task-oriented training for recovery after stroke. The number of repetitions needed is suggested to be in the order of hundreds, but this is not currently being achieved in clinical practice. In an effort to bridge this evidence-practice gap, we have developed a behaviour change intervention that aims to increase provision of upper limb repetitive task-oriented training in stroke rehabilitation. This paper aims to describe the systematic processes that took place in collaboratively developing the behaviour change intervention.

**Methods:**

The methods used in this study were not defined *a priori* but were guided by the Behaviour Change Wheel. The process was collaborative and iterative with four stages of development emerging (i) establishing an intervention development group; (ii) structured discussions to understand the problem, prioritise target behaviours and analyse target behaviours; (iii) collaborative design of theoretically underpinned intervention components and (iv) piloting and refining of intervention components.

**Results:**

The intervention development group consisted of the research team and stroke therapy team at a local stroke rehabilitation unit. The group prioritised four target behaviours at the therapist level: (i) identifying suitable patients for exercises, (ii) provision of exercises, (iii) communicating exercises to family/visitors and (iv) monitoring and reviewing exercises. It also provides a method for self-monitoring performance in order to measure fidelity. The developed intervention, PRACTISE (Promoting Recovery of the Arm: Clinical Tools for Intensive Stroke Exercise), consists of team meetings and the PRACTISE Toolkit (screening tool and upper limb exercise plan, PRACTISE exercise pack and an audit tool).

**Conclusions:**

This paper provides an example of how the Behaviour Change Wheel may be applied in the collaborative development of a behaviour change intervention for health professionals. The process involved was resource-intensive, and the iterative process was difficult to capture. The use of a published behaviour change framework and taxonomy will assist replication in future research and clinical use. The feasibility and acceptability of PRACTISE is currently being explored in two other stroke rehabilitation units.

**Electronic supplementary material:**

The online version of this article (doi:10.1186/s13012-015-0223-3) contains supplementary material, which is available to authorized users.

## Background

The evidence base for stroke rehabilitation has grown exponentially in recent years. There is now strong evidence supported by high-quality trials, and underpinned by motor learning and neuroplasticity literature [1], to support intensive repetitive task-oriented training for recovery after stroke [2]. After a stroke, two thirds of survivors will achieve independent ambulation but less than half will recover upper limb function at 6 months [3]. Upper limb impairment after stroke has been shown to significantly influence quality of life [4] but remains a critical and neglected area of stroke rehabilitation [5]. A positive relationship has been found between the time scheduled for therapy and recovery, suggesting that increased doses of therapy may lead to clinically meaningful improvements [6]. However, the optimal dose of therapy in stroke rehabilitation is not yet known, with current guidance recommending that stroke survivors have as much opportunity as possible to repeatedly practise upper limb tasks [7]. Neuroplasticity literature suggests that repetitions in the order of hundreds are likely to be necessary [8] to maximise recovery after stroke. This contrasts starkly with clinical practice, where it has been reported that the average amount of time spent treating the upper limb in therapy sessions is between 0.9 and 7.9 min [9] resulting in, on average, just 32 repetitions per session [10].

There exists a clear evidence-practice gap in stroke rehabilitation with methods to increase the amount of repetitive task-oriented training for the upper limb urgently needed. GRASP (Graded Repetitive Arm Supplementary Program) is a self-directed arm and hand exercise programme that was developed by researchers at the University of British Columbia. It is an evidence-based intervention that aims to address this gap and increase the numbers of repetitions that stroke survivors complete during rehabilitation [11]. The stroke survivor is taught by a therapist how to complete a range of upper limb exercises included in the GRASP manual and then completes the exercises outside of their therapy time, with the help of a family member or carer where possible. GRASP has experienced unusually rapid uptake into clinical practice. For example, despite only being published in 2009, and not being explicitly recommended in the UK stroke guidelines, approximately 63% of UK therapists who responded to a survey were aware of GRASP by 2013, of whom 23% had used GRASP and 11% were regular users [12]. In order to explore the reasons for this rapid uptake, a formative evaluation of the implementation of GRASP in British Columbia, Canada was carried out [13]. Therapists working in stroke rehabilitation reported that key factors in finding out about the intervention were their own personal networks with colleagues from academia and clinical practice, and the free online availability of GRASP. A notable finding from this evaluation was that although the uptake of GRASP was good, key components of the intervention were modified when implemented by therapists in routine clinical practice. For example, GRASP was provided to non-stroke patients (e.g. spinal cord injury, brain injury patients); the exercises were often provided separately as opposed to providing the full manual, and the dose, when monitored, was less than the recommended amount.

Low implementation fidelity has been reported in previous trials in stroke rehabilitation, e.g. the training caregivers after stroke (TRACS) trial [14]. This was a cluster randomised controlled trial (RCT) involving 36 stroke units assigned to either an intervention to promote stroke carer training or usual care. The intervention was targeted at routine multidisciplinary stroke staff and included multiple components: carer resources (training manual and training record), taught sessions for staff and staff resources (slides and recorded staff training sessions). It was designed to be cascaded by trained staff to those not yet trained to incorporate the intervention into usual care. However, a process evaluation published in this journal demonstrated poor implementation, with no mechanisms existing for ensuring fidelity of the intervention in practice [15]. Results at 6 months demonstrated no clinical or statistical differences between groups on the primary outcomes of functional independence of patients or caregiver burden. The authors highlighted the need for the development of systems to monitor intervention use within practice and for researchers to consider implementation strategies *a priori*, ideally in partnership with the end users of the intervention.

Rehabilitation interventions tend to be complex interventions, i.e. interventions comprising several components acting either independently or interdependently [16]. Successful implementation of complex interventions, such as GRASP or TRACS, relies on changing the behaviours of those responsible for their implementation [16]. However, consideration of behaviour change of healthcare professionals in the development and implementation of complex interventions has traditionally been given cursory attention. Fewer still formally test the feasibility of proposed interventions prior to evaluation [17]. Developing behaviour change interventions, which by definition are complex, is a growing field of enquiry, but as of yet, there is no gold standard method reported within the literature. Guidance, such as the MRC framework for the development and evaluation of complex interventions [16], identifies the use of theory as a fundamental component of intervention development, but how theories should be used to inform methods is less clear.

One way to enhance the development of interventions targeting health professionals’ behaviour could be through applying the framework outlined in the Behaviour Change Wheel (BCW) [18]. This was first detailed in a publication in 2011, with further information in a book which was published following the commencement of this project [19]. The BCW aims to provide a systematic process from behavioural analysis to intervention design. It was developed following a systematic review and a synthesis of 19 existing frameworks of behaviour change. It has three layers; at its core, the COM-B (capability, opportunity, motivation and behaviour) model was surrounded by nine intervention functions and seven policy categories. The authors tested the reliability of the framework against two existing policies for tobacco and obesity management. However, the process of using the BCW to develop new interventions and the extent to which applying the BCW will lead to more successful interventions has yet to be evaluated.

Using the BCW as a guide, we have developed a behaviour change intervention that aims to increase intensity of upper limb exercise in stroke rehabilitation. Many challenges exist both in devising the content of behaviour change interventions and in systematically reporting interventions in a level of detail sufficient to allow replication in other studies or for use in practice [20]. The aim of this paper is to describe the processes that took place in developing a behaviour change intervention and to describe the resulting intervention. This will contribute to the growing evidence base on the development of complex interventions and allow for improved interpretation of findings in future studies testing the effectiveness of the intervention.

## Methods

**Figure 1:**
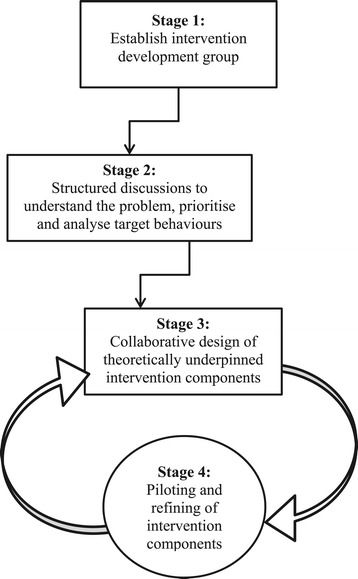
**Stages of development.**

We did not define the methods for developing the behaviour change intervention *a priori*. It was an iterative process that was guided, though not rigidly, by the BCW [18]. The stages of development that emerged during the process are illustrated in Figure 1. As there was no change in treatments provided to patients from accepted standards, ethical approval from the National Research Ethics Service (NRES) was not required for this phase of the study. Approval was obtained from the local Research and Development (R&D) office for the site.

### Stage 1: Establish intervention development group

Stage one entailed establishing an intervention development group that engaged key stakeholders and end users of the intervention, as well as researchers. This sustained collaborative approach to intervention development is novel as rehabilitation interventions have traditionally been developed by academic research teams for the purposes of testing in efficacy trials with limited on-going practitioner input. However, as there is now a robust evidence base underpinning the importance of intensity in stroke rehabilitation, the focus of research has shifted from efficacy studies to translational research to implement this evidence in practice. The rationale for the collaborative partnership was to maximise the “potential fit” of the developed intervention, and intervention materials, with the context in which the intervention would be implemented [21].

### Stage 2: Structured discussions to understand the problem, prioritise target behaviours and analyse target behaviours

In stage two, the intervention development group engaged in structured discussions to understand the problem (i.e. how upper limb exercise could be increased in the stroke rehabilitation unit) and identify behaviours that could be amenable to change within the limits of the project using the BCW [18] as a guide and to prompt discussion. Information from formative research on current UK therapy practice for prescribing upper limb exercises [22], and how the GRASP has been previously implemented [13], was used as a basis for brainstorming possible target behaviours. A list of all potential target behaviours was generated by the group, which the group then prioritised according to how amenable to change they perceived them to be using guidance from the BCW.

Each target behaviour was analysed to determine how best behaviour change could be achieved using the COM-B model, the hub of the BCW [19]. COM-B is a simple model to understand behaviour based on capability (psychological or physical ability to enact the behaviour), opportunity (the physical and social environment that enables the behaviour) and motivation (reflective and automatic mechanisms that activate or inhibit behaviour). A definition of the COM-B model and previous examples were given to the group to facilitate discussions to identify what needed to change in order for therapists to be able to perform these target behaviours.

### Stage 3: Collaborative design of theoretically underpinned intervention components

The methods in stage three were less aligned with the Behaviour Change Wheel. It entailed a collaborative design exercise to identify intervention components and was informed by the Behaviour Change Technique Taxonomy (v1) (BCTTv1) [23]. The aim of the BCTTv1 is to provide a reliable and systematic method of describing and categorising behaviour change techniques (BCTs) used in interventions. Employing consistent terminology to describe these techniques allows developers to identify the active ingredients of interventions, test these active ingredients and comprehensively describe interventions to facilitate replication in future research [24]. During the design exercise, the BCTTv1 [23] was used by the intervention development group to facilitate discussion around potential behaviour change techniques, and delivery methods, to ensure all options were considered. A description of what intervention components could look like was then drafted. The research team produced versions of the intervention components which were presented back to the therapists for additional feedback and refining.

### Stage 4: Piloting and refining of intervention components

Stage four represents the on-going reflexive cycle during which the developed intervention components were piloted and refined based on the experiences of the end users. Between development meetings, the therapy team had the opportunity to test each of the intervention components designed in the real-life clinical setting for a few weeks. At each development meeting, feedback was obtained and discussed, and the reflexive cycle repeated.

## Results

### Stage 1: Establish intervention development group

The intervention development group comprised a collaborative partnership between two members of the research team (LC and NM) and a local therapy team (physiotherapists, occupational therapists, rehabilitation assistants) representative of the end users of the intervention. The site where intervention development took place was originally identified through existing contacts between LC and local therapists working in stroke rehabilitation. The site was a stroke rehabilitation unit in a conurbation in the north west of England, located separately to the acute stroke unit. LC and NM lead the intervention development group meetings. Both are female chartered physiotherapists working full time in the field of stroke rehabilitation research and implementation science. All members of the therapy team were invited to attend meetings and attendance ranged from 4 to 8 staff members at each meeting. A senior physiotherapist (NHS Band 6) with 4-year rotational experience of working in stroke rehabilitation took the lead on keeping the rehabilitation team informed about the development process and progress and ensuring that the intervention documentation was being completed. Meetings took place at the development site at times deemed suitable by the therapy team. In total, eight meetings were held over a period of 7 months.

### Stage 2: Structured discussions to understand the problem, prioritise target behaviours and analyse target behaviours

**Figure 2:**
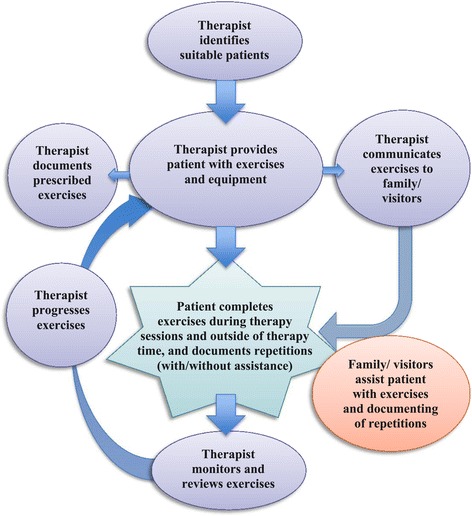
**The interdependent network of behaviours.**

The problem to be addressed was the intensity of upper limb task-oriented training completed by stroke survivors in the stroke rehabilitation unit. Structured discussions during intervention development group meetings highlighted a range of different interdependent behaviours that need to be performed to bring about this increased intensity of exercise. These are illustrated in Figure 2.

Based on the BCW method for prioritising target behaviours, it was decided by the intervention development group that therapist level behaviours would be the focus of the intervention in this study for the following reasons: (i) they were viewed as the first steps in the causal chain and hence have spillover effect, (ii) they were considered by the group to be amenable to change and (iii) following piloting demonstrated strong potential to positively increase upper limb exercise in the stroke rehabilitation unit.

The group prioritised four target behaviours at the therapist level:

1. Identifying suitable patients for exercises.
2. Provision of exercises and equipment.
3. Communicating exercises to family/visitors.
4. Monitoring and reviewing exercises.

**Figure 3:**
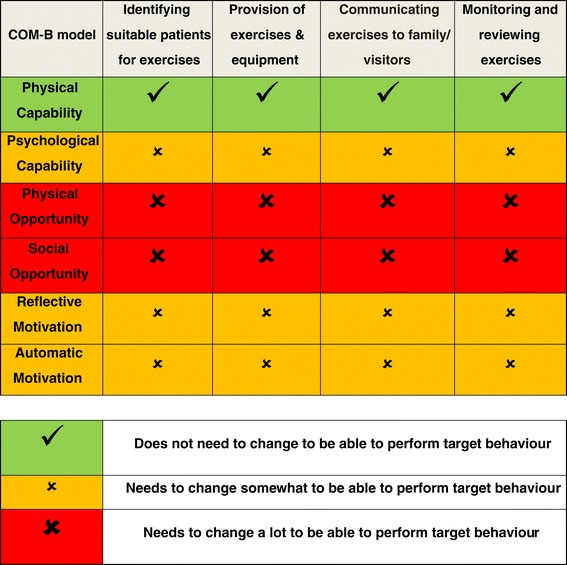
**Behavioural analysis for the four target behaviours using the COM-B model**

Each target behaviour was analysed to determine what needed to change and how best behaviour change could be achieved using the COM-B model, the hub of the BCW. The views of the therapists discussed here were also corroborated with formative research findings and other relevant literature. The results of the behavioural analysis for the target behaviours using the COM-B model are shown in Figure 3.

#### Capability

- All therapists had the physical capability to perform the four target behaviours. Therapists discussed that from assessments, it was not always clear who should be prescribed upper limb exercises, in particular who should be prescribed exercises to be practised outside of therapy time. Therapists also discussed that entry level therapists new to stroke rehabilitation can often find it difficult to identify exercises suitable to the stroke survivor’s level of ability. All therapists not only discussed the importance of family and carer involvement in rehabilitation but also highlighted the challenge of effectively engaging families/visitors in the rehabilitation process.

#### Opportunity

- The most frequently discussed issue that needed to be addressed in order for therapists to successfully perform the four target behaviours was the limited time available to them in their working day. Therapists also discussed issues around social opportunity such as the limited amount of emphasis placed on addressing upper limb impairment in inpatient stroke rehabilitation settings compared to that placed on recovery of the lower limb, transfers and mobilising. In inpatient stroke rehabilitation, there is also limited physical opportunity for therapists to monitor, review and progress prescribed exercises prior to discharge as a result of continually reducing length of stay and early discharge of stroke survivors.

#### Motivation

- Limited physical and social opportunity to perform the target behaviours was identified as having an effect on therapist’s motivation. All therapists discussed the importance of increasing intensity of upper limb exercises in inpatient stroke rehabilitation settings, and their desire to more actively engage in this, but the lack of external drivers hampered this motivation. For example, currently, in the national stroke rehabilitation guidelines, there are targets for the time taken until assessment and amount of therapy received but no quantifiable targets relevant to upper limb rehabilitation [7].

### Stage 3: Collaborative design of theoretically underpinned intervention components

As illustrated in the behavioural analysis (Figure 3), physical opportunity and social opportunity emerged as the domains most in need of change to facilitate therapists in performing the target behaviours. To manage the scale, and the scope of the intervention, the intervention development group focused on developing intervention components, underpinned by behaviour change techniques from the Behaviour Change Technique Taxonomy (v1) that could address these domains.

The developed intervention is PRACTISE (Promoting Recovery of the Arm: Clinical Tools for Intensive Stroke Exercise). PRACTISE consists of team meetings and the PRACTISE Toolkit.

1. Team meetings.

Although the face-to-face meetings between the clinical team and research teams at this stage in the study were initially required for the development process, they were ultimately identified as an important component of the intervention itself as they ensured commitment to implementation and provided opportunity for self-monitoring of behaviour (i.e. measuring fidelity to the developed intervention), problem solving and action planning.

2. PRACTISE Toolkit.

Please see Additional file 1: The PRACTISE Toolkit for draft versions of each of the following components. The developed toolkit included:

- A screening tool and upper limb exercise plan: to enable therapists to efficiently identify patients that should be receiving upper limb exercises and document prescribed upper limb exercises in the medical notes.
- A PRACTISE exercise pack: to enable therapists to efficiently communicate (verbally and in written format) the rationale for the exercise programme, the individual exercises (based on GRASP exercises, with written and pictorial instructions) to be completed by the patient, and to enable the therapist and the patient to monitor repetitions of exercises using an exercise diary.
- An audit tool: to enable therapists to self-monitor performance around provision of upper limb exercises to suitable patients in the stroke rehabilitation unit.

Data from the screening tool and the upper limb exercise plan were used as a source of information for the audit tool to monitor the numbers of appropriate patients in the unit for which the target behaviours were being performed (see Additional file 1: The PRACTISE Toolkit). It should be noted that the components of the toolkit are intended to have some flexibility in terms of form (e.g. to fit with local systems/policies), but the intervention aim and BCTs are standardised. The intervention components, their underpinning behaviour change techniques and what they aimed to change are summarised in Table 1.

**Table 1:** **Intervention components, underpinning behaviour change techniques and what they aimed to change**

**What needed to change**		**Behaviour change techniques**	**Intervention components (see Additional file** **1** **)**
Physical opportunity	Due to time constraints, more efficient ways of performing the target behaviours were needed	4.1 Instruction on how to perform the behaviour 3.2 Social support (practical)	2a. Screening tool and exercise plan 2b. PRACTISE pack
Social opportunity	Getting upper limb rehabilitation higher up on the agenda was needed through managerial support and team engagement	1.2 Problem solving 1.4 Action planning 1.9 Commitment 2.3 Self-monitoring of behaviour	1. Team meetings 2c. Audit tool

### Stage 4: Piloting and refining of intervention components

As the intervention components were drafted, they were pilot tested by the therapy team. Pilot testing allowed the group to establish whether or not the intervention impacted on the prioritised behaviours in the desired way and also to establish in what way, if any, the intervention components could be refined and improved. Following piloting, all components stayed, but the format was often modified or refined. An example of this was the introduction of the “Front sheet” (see Additional file 1: The PRACTISE Toolkit). Originally, the “Exercise Plan” was provided to patients as part of their PRACTISE Pack. However, feedback from therapists piloting the form suggested that it contained too much information and was too complicated for this purpose. The simpler “Front sheet” was developed to include the patient’s goal, the exercises they had been prescribed, the names of individuals willing to assist with the exercises and the date for review.

## Discussion

This paper describes our experience of developing a complex behaviour change intervention that aims to increase upper limb repetitive task-oriented training in stroke rehabilitation units. The developed intervention is PRACTISE. PRACTISE consists of team meetings and the PRACTISE Toolkit (a screening tool and upper limb exercise plan, PRACTISE exercise pack and an audit tool).

Developing and describing PRACTISE was resource-intensive. The efforts required for intervention development have been noted previously [14,25], yet securing funding and publishing this type of work is still problematic. In this study, we used the Behaviour Change Wheel, although not rigidly, to guide the development process [19]. In trying to document the iterative process and maintain clarity, it is presented as more linear than it actually was. Although the BCW did provide a framework, there are still many ways in which it could be applied. In previous research studies applying the BCW to design an intervention, researchers have used interviews and questionnaires with the target group to identify factors that need to change in order for behaviour to occur [26-28]. We built on formative research findings [13] and had structured discussions with the intervention development group. As the development process was over several months, therapists were able to reflect on the discussions over time and consider the behavioural analysis during their clinical practice and could still input these into the development. This method has the limitations of being arguably less methodologically robust (e.g. not transcribed/less reproducible) but meant that it was insightful and comprehensive. The fact that it built on formative research which involved therapists both in the UK and Canada provides some credence that the findings will be generalizable to other stroke rehabilitation units.

The collaborative design of the intervention components (stage 3) was less aligned with the Behaviour Change Wheel. Based on the behavioural analysis, any of the BCW intervention functions could be selected and hence any of the policy categories. In addition, the policy categories are not well defined and, as the name suggests, aimed more at a policy level (e.g. legislation, fiscal measures). Our intervention would all fit under the category “service provision,” which incorporates a vast array of potential intervention components. It was therefore felt the BCW was less directive and helpful at this stage. However, the Behaviour Change Technique Taxonomy (v1) was particularly useful during the intervention development process as it provided common terminology to describe the purpose of the intervention components. This aligns with previous evidence suggesting that when developing and testing complex interventions, there is a need to be clear about the function of the intervention components but to allow some flexibility with the form to allow adaptation at the local context [29]. Using specified behaviour change techniques from a published taxonomy, together with the embedded toolkit performance measures, will provide components through which fidelity to the intervention can be measured in future research and clinical use. It is anticipated that this will assist with the difficult process of unpicking the active mechanisms within the intervention during an evaluation study, with the importance of undertaking this careful development work recognised [30].

We also had the opportunity to test and refine developed intervention components with the end users. This stage is not included in the BCW but emerged as key to our development process. Involving users has been demonstrated to be the best predictor for ensuring research is translated into practice [31], and so, in this study, we endeavoured to maximise the acceptability of the intervention through collaborative working with users, i.e. stroke therapy teams. They reported that the developed toolkit components were inexpensive, acceptable to the therapy team and fitted well with current methods of documentation, and were practical for therapists and patients/families.

Despite the fact that we did not define the development process in advance, numerous similarities can be seen between our methods and research in both the implementation science literature and other areas such as quality improvement, e.g. Plan-Do-Study Act (PDSA) cycles. It is often the same premise (i.e. understand, test, evaluate, refine), understood and explored using different theories or frameworks. Describing our development process should be of interest to others who are trying to develop interventions to change behaviour of health professionals.

### Future work

Following the development and testing of PRACTISE, the intervention now needs to be tested in other stroke rehabilitation units prior to a definitive effectiveness trial, both in terms of change in health professional behaviour and patient outcomes. A feasibility case study of the PRACTISE toolkit in two stroke rehabilitation units is currently on-going.

## Conclusion

This paper provides an example as to how the Behaviour Change Wheel may be applied in the collaborative development of a behaviour change intervention for health professionals. The process involved was resource-intensive, and it was difficult to capture the iterative process. The use of the Behaviour Change Wheel and behaviour change techniques from a published taxonomy provide an example of how these frameworks may be applied and will assist replication in future research and clinical use.

## Additional file

Additional file 1:
**Practise Toolkit.** Draft version of the PRACTISE Toolkit.
