# Supplementary material for: Development of a behaviour change intervention to increase upper limb exercise in stroke rehabilitation
Source: Implement Sci. 2015 Mar 12;10:34. doi: 10.1186/s13012-015-0223-3 (PMC4358857; doi:10.1186/s13012-015-0223-3)
Supplement: Additional file 1: — Practise Toolkit. Draft version of the PRACTISE Toolkit. [file 13012_2015_223_MOESM1_ESM.docx]

**Screening Tool**

The purpose of this screening form is to help identify stroke patients that are suitable to complete upper limb exercises, with or without assistance/ modifications. All patients that present with upper limb motor impairment should be screened for suitability. The category under which they fall under, whether or not they have been prescribed additional exercise and the reasons should be documented.

**GREEN: Prescribe exercises to be completed independently/with assistance (family, rehabilitation assistant)**

**[consider GRASP Manual]**

**NO**

**YES**

**AMBER: Prescribe exercises to be completed with assistance (family, rehabilitation assistant); and modified as needed to address shoulder pain/tone.**

**NO**

**RED: Not suitable for additional exercise**

**NO**

**NO**

**Additional exercise not indicated**

**Does the patient have:**

1. **Difficulty understanding**
2. **Difficulty communicating**
3. **Poor safety awareness**
4. **Low motivation**
5. **Painful shoulder, 🡩 tone**

**YES**

**Can the patient shoulder shrug and have they a flicker of wrist/finger extension on their affected side?**

**YES**

**YES**

**Does the patient have an upper limb goal?**

**Does the patient have upper limb motor impairment?**

**YES**

**YES**

**YES**

**YES**

**Upper Limb**

Patient Information:

**Exercise Plan**

| **RAG category:** | Amber 🞎 Green 🞎 |
| --- | --- |
| **Current upper limb function:** |  |
| **Goal:** |  |
| **Types of exercises needed to achieve goal:** | ROM 🞎  Strengthening 🞎  Task-specific + fine motor 🞎 |
| **Prescribed exercises:** | **1.** |
|  | **2.** |
|  | **3.** |
|  | **4.** |
|  | **5.** |
| **Key individual(s) that have agreed to support patient with exercises:**  (provide name, relation to patient and date educated on exercises) |  |
| **Potential issues:** |  |
| **Review date:** |  |

Therapist: ________________________________


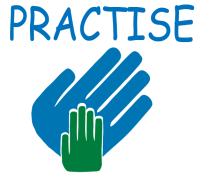


**www.practiseproject.com**

Date: ______________

**PRACTISE Pack: Front Sheet**


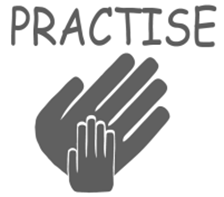


**PRACTISE PACK**

**Name:**

**Goal:**

**Exercises:**

**1.**

**2.**

**3.**

**5.**

**4.**

**People available to assist with exercises:**

**Review date (within two weeks): __/__/__**

**Therapist signature: ___________________ Date: ______**

**PRACTISE Pack: Exercise Sheet (example)***

*Adapted with permission from the Graded Repetitive Arm Supplementary Program (GRASP) manuals


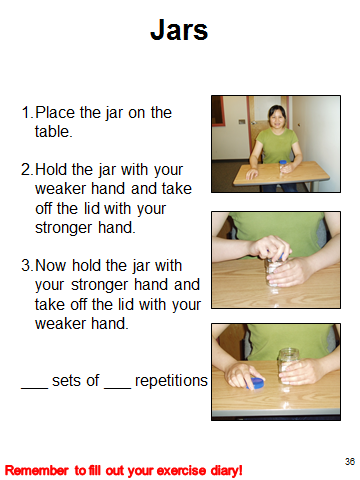


**PRACTISE Pack: Exercise Reminder and Diary**

To help your arm to get better you need to **practice** doing things that you find hard


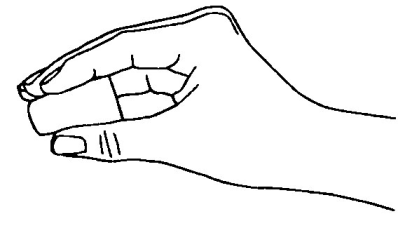


You have been given these **arm** **exercises** to practice

The **number of times** you should do each exercise has been written on each page

**1 2 3**


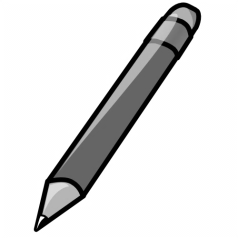


**Write down** how many times you do each exercise so you can keep track of how much you are able to do


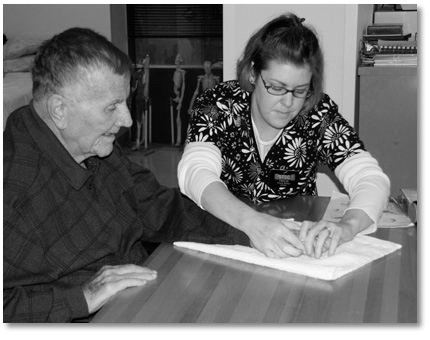


Try to practice your exercises with **somebody that can help you!**


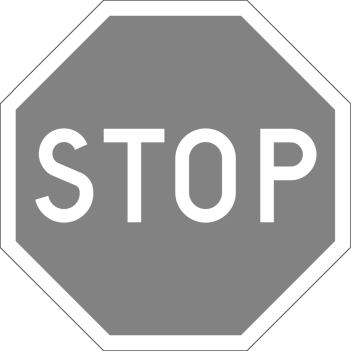


If you have **PAIN** **☹** stop doing the exercises and tell your named therapist.

_________________________

To keep track of how much exercise you do, please write down the number of exercises you do every day. There is space to write the date and for you (or the person that helped you) to initial the box.

| Date | |  | |  | |  | |  | |  | |  | |  | |
| --- | --- | --- | --- | --- | --- | --- | --- | --- | --- | --- | --- | --- | --- | --- | --- |
| Exercise | | No. | Initial | No. | Initial | No. | Initial | No. | Initial | No. | Initial | No. | Initial | No. | Initial |
| Example | Pouring | 20 MB | | 25 MB | | 20 MB | | 30 MB | | 20 MB  20 MB | | 30 MB | | 30 MB | |
| 1 |  |  | |  | |  | |  | |  | |  | |  | |
| 2 |  |  | |  | |  | |  | |  | |  | |  | |
| 3 |  |  | |  | |  | |  | |  | |  | |  | |
| 4 |  |  | |  | |  | |  | |  | |  | |  | |
| 5 |  |  | |  | |  | |  | |  | |  | |  | |
| Total | |  | |  | |  | |  | |  | |  | |  | |

**If you are initialling this form on behalf of the patient, please complete below so we can keep track of who is helping.**

e.g. MB Matthew Brown Son.

Named Therapist: ____________________________

Review Date: ________________________________

| **Pt. Name** | **RAG^1^** | **Exercise Plan completed** | | **Goal** | **Exercises sheets provided** | | **Family/carer/ visitors informed** | | **Date of review** | **Reviewed + Progressed** | | **Named Therapist** |
| --- | --- | --- | --- | --- | --- | --- | --- | --- | --- | --- | --- | --- |
|  |  | Yes | No |  | Yes | No | Yes | No |  | Yes | No |  |
|  |  |  |  |  |  |  |  |  |  |  |  |  |
|  |  |  |  |  |  |  |  |  |  |  |  |  |
|  |  |  |  |  |  |  |  |  |  |  |  |  |
|  |  |  |  |  |  |  |  |  |  |  |  |  |
|  |  |  |  |  |  |  |  |  |  |  |  |  |
|  |  |  |  |  |  |  |  |  |  |  |  |  |
|  |  |  |  |  |  |  |  |  |  |  |  |  |
|  |  |  |  |  |  |  |  |  |  |  |  |  |

**Audit Tool**

^1^RAG stands for Red/Amber/Green categorised using the screening tool and represents the target behaviour of identifying suitable patients for upper limb exercises

**Audit Tool (Example of data presented in excel spreadsheet)**

|  |  |  |  |  |  |  |
| --- | --- | --- | --- | --- | --- | --- |
|  | **Jun-14** | | | | | |
|  |  |  |  |  | **Number** | **%** |
|  | Number of new stroke admissions | | | | 10 | 42% |
|  | Number of stroke patients through unit | | | | 22 |  |
|  | Number screened as Red | | | | 11 | 50% |
|  | Number screened as Amber | | | | 6 | 27% |
|  | Number screened as Green | | | | 0 | 0% |
|  | **AMBER** | | | | | |
|  |  | | | **Needed** | **Received** | **%** |
|  | Number of patients that had exercise plan completed | | | 6 | 5 | 83% |
|  | Number of patients exercises prescribed | | | 6 | 5 | 83% |
|  | Number of patients that had exercises communicated to family/carers/visitors | | | 6 | 5 | 83% |
|  | Number of patients reviewed and progressed | | | 6 | 5 | 83% |
|  | **GREEN** | | | | | |
|  |  | | | **Needed** | **Received** |  |
|  | Number of patients that had exercise plan completed | | | 0 |  |  |
|  | Number of patients exercises prescribed | | | 0 |  |  |
|  | Number of patients that had Next of Kin informed | | | 0 |  |  |
|  | Number of patients reviewed | | | 0 |  |  |
|  | Number of patients reporting positive experience | | | 0 |  |  |
|  | Number of patients referred to community neuro team | | | 0 |  |  |
|  |  |  |  |  |  |  |
| \|  \| \| --- \| |  |  |  |  |  |  |
